# Association between molecular markers and behavioral phenotypes in the immatures of a butterfly

**DOI:** 10.1590/1678-4685-GMB-2017-0073

**Published:** 2018-03-19

**Authors:** Janaína De Nardin, Vanessa Buffon, Luís Fernando Revers, Aldo Mellender de Araújo

**Affiliations:** 1 Programa de Pós-Graduação em Genética e Biologia, Departamento de Genética, Universidade Federal do Rio Grande do Sul, Porto Alegre, RS, Brazil; 2 Laboratório de Genética Molecular Vegetal, Centro Nacional de Pesquisa de Uva e Vinho, Empresa Brasileira de Pesquisa Agropecuária (EMBRAPA) Uva e Vinho, Bento Gonçalves, RS, Brazil

**Keywords:** Kin discrimination, caterpillar-egg cannibalism, Lepidoptera, *Heliconiu*s, AFLP

## Abstract

Newly hatched caterpillars of the butterfly *Heliconius erato phyllis* routinely cannibalize eggs. In a manifestation of kin recognition they cannibalize sibling eggs less frequently than unrelated eggs. Previous work has estimated the heritability of kin recognition in *H. erato phyllis* to lie between 14 and 48%. It has furthermore been shown that the inheritance of kin recognition is compatible with a quantitative model with a threshold. Here we present the results of a preliminary study, in which we tested for associations between behavioral kin recognition phenotypes and AFLP and SSR markers. We implemented two experimental approaches: (1) a cannibalism test using sibling eggs only, which allowed for only two behavioral outcomes (cannibal and non-cannibal), and (2) a cannibalism test using two sibling eggs and one unrelated egg, which allowed four outcomes [cannibal who does not recognize siblings, cannibal who recognizes siblings, “super-cannibal” (cannibal of both eggs), and “super non-cannibal” (does not cannibalize eggs at all)]. Single-marker analyses were performed using χ^2^ tests and logistic regression with null markers as covariates. Results of the χ^2^ tests identified 72 associations for experimental design 1 and 73 associations for design 2. Logistic regression analysis of the markers found to be significant in the χ^2^ test resulted in 20 associations for design 1 and 11 associations for design 2. Experiment 2 identified markers that were more frequently present or absent in cannibals who recognize siblings and super non-cannibals; i.e. in both phenotypes capable of kin recognition.

## Introduction

The evolution of morphological, behavioral and life history traits is underpinned by the evolution of a large number of loci (Lynch and Walsh, 1998; Erickson *et al.*, 2004). Quantitative traits are under polygenic control. As a consequence, they frequently show continuous variation within and between populations (Falconer and Mackay, 1996); however, as in the case of threshold traits, their phenotypic variation does not need to be linear (Roff *et al.*, 1999). Evolutionary biologists have sought to examine the genetic basis of these traits. One approach relies on the use of molecular markers to identify quantitative trait loci (QTLs), i.e. genetic loci that contribute to quantitative traits (Lynch and Walsh, 1998; Erickson *et al.*, 2004).

Genetic mapping of QTLs has become a routine tool to study plants, animals and humans. The available methods fall into two main categories that are based on related genetic principles: linkage analysis and association studies (Olson *et al.*, 1999; Wu *et al.*, 2002). Here we use a study design that relies on association analysis. Genetic association studies are designed to identify genetic loci where allelic states are correlated with the phenotype of interest. The associations of interest are causal and identify loci whose different alleles have different effects on the phenotype. However, even if the causal locus itself is not genotyped it may be possible to identify it indirectly through association with genotyped loci that are located nearby in the genome (Astle and Balding, 2009). A recent advance is genome-wide association analysis, in which a large number of single locus tests are performed to examine marker loci covering the entire genome for association with the phenotype (Bush and Moore, 2012). Each single-marker test assesses the segregation of a phenotype with respect to the marker genotype, indicating which markers are associated with the phenotypic trait of interest and pointing to the existence of potential QTLs in the genomic neighborhood of associated markers (Doerge, 2002).

Lepidoptera is a diverse clade that has long attracted the attention of biologists interested in ecological and evolutionary processes, from the classical studies of Edmund Ford (Ford, 1964, 1975) on *Maniola jurtina* and other butterflies and moths, to Kettlewell’s experiments (Kettlewell, 1955) on selection in industrial melanism, until recent studies on butterflies of the genus *Heliconius* (Merrill *et al.*, 2015).

*Heliconius* butterflies are a well-established model for studies of ecology, natural selection and speciation (Brown Jr, 1981; Jiggins *et al.*, 2005; Joron *et al.*, 2011). As the most widespread species of the genus, *Heliconius erato* is present in many habitats and forest types, from Mexico to northern Argentina. Of all subspecies, *H. erato phyllis* has the widest geographical distribution, as well as greatest environmental tolerance (Ramos and Freitas, 1999).

The newly hatched caterpillars of *H. erato phyllis* routinely cannibalize neighboring eggs. While the eggs of both sibling and unrelated individuals can be preyed upon, sibling eggs are cannibalized significantly less frequently than unrelated eggs (De Nardin and Araújo, 2011). This is an example of kin recognition, which can be strictly defined as the ability to identify a relationship as identical by descent (Hamilton, 1964a,b; Breed, 2014). In a recent study published by our group, we found a genetic component to the non-cannibalistic behavior, a characteristic associated with the recognition of relatedness (De Nardin *et al.*, 2017). We observed that the frequency of non-cannibalism increases in offspring of inbred crosses. Cannibalistic behavior is thought to be influenced by several genes, or QTLs, and the assumption is that the manifestation of a non-cannibalistic phenotype depends on a threshold for their joint expression. Here, we present a preliminary study, in which we explored molecular marker genotyping strategies in order to search for associations between egg-cannibalism as a kin recognition behavioral phenotype, and AFLP and SSR alleles and genotypes.

## Material and Methods

### Butterflies and experimental design

*Heliconius erato phyllis* females were captured from 2011 to 2014 in nine wild populations. These were distant from each other by at least 2 km until 160 km, and all of them were located in the state of Rio Grande do Sul, Brazil. Females were maintained in open air insectaries measuring approximately 3 x 2 x 2 m (length x width x height) at the Department of Genetics, Universidade Federal do Rio Grande do Sul, Porto Alegre, RS, Brazil. Females of this species are monandric, so that eggs laid by a single female are certain to be full siblings. The experimental design followed De Nardin and Araújo (2011). The females had already mated in the wild. Eggs were collected daily with the aid of brushes and placed at the vertices of an equilateral triangle made of green paper cardboard with a side length of 0.5 cm. The triangles were kept at room temperature in Petri dishes (8 cm in diameter and 1.5 cm in height) and covered with a lightly moistened paper towel to prevent dehydration of the eggs. Upon hatching of the first caterpillar, the presence/absence of cannibalism toward the remaining eggs was observed over a 45 minute period. We employed two experimental designs:

1. Tests with sibling eggs only. In this case, whenever cannibalism occurred, the caterpillar had either not recognized the sibling egg, or was hungry enough to cannibalize it anyway (evidence from previous experiments indicated that there was kin recognition). This behavioral phenotype was represented as C. The absence of cannibalism likely meant that the caterpillar had recognized the egg as being that of a sibling; this phenotype was represented as NC.
2. Tests with two sibling eggs and a non-related egg. These tests were valid for our purposes if the first egg to hatch was one of the siblings. In this case, the following phenotypes were possible: cannibalism of a sibling egg (no sibling recognition, represented as CNR), cannibalism of a non-related egg (sibling recognition; CR), cannibalism of both eggs (“super cannibal”; SC), and finally, no cannibalism at all (“super non-cannibal”; SNC).

Immatures hatched as part of these experiments were reared in the laboratory at a controlled temperature (25 °C) and were fed daily with *Passiflora suberosa* until they reached the adult stage. Adults hatched in experiment 1 were subjected to either outbred or inbred crosses, as described in the following. Outbred crosses: FNC x MC (four repeats), FC x M NC (four repeats), FC x MC (two repeats), and FNC x MNC (two repeats). Inbred crosses: FC x M C (two repeats), FNC x MNC (three repeats) and FNC x MC (one repeat). Adults hatched in experiment 2 were subjected to outbred crosses, as follows: FCR x MCNR (one repeat), FCNR x MNCS (two repeats), and FCS x MCS (one repeat). Finally, the egg-cannibalism experiments were repeated with the eggs from these crosses. Eggs of caterpillars hatched as part of experiment 1 were subjected to experimental design 1, eggs of caterpillars hatched as part of experiment 2 were subjected to experimental design 2.

### Molecular markers

Total DNA for genotyping was extracted from adult individuals following the protocol described by Mega and Revers (2011) and diluted to a concentration of 50 ng/μL for use in AFLP and SSR assays.

AFLPs markers were obtained from the AFLP Plant Mapping Protocol (Applied Biosystems, P/N 4303146F). Adapter ligation and pre-selective amplification were performed using the AFLP® Ligation and Preselective Amplification Module for Small Genomes (Applied Biosystems, Foster City, CA, USA) and *Eco*RI and *Mse*I restriction enzymes. Selective amplification was performed using the AFLP® Selective Amplification Primers (Applied Biosystems), using 12 primer pair combinations: *Eco*RI-TA-JOE (green fluorescence)/*Mse*I-CAA, *Eco*RI-TA-JOE/*Mse*I-CAC, *Eco*RI-TA-JOE/*Mse*I-CTG, *Eco*RI-TA-JOE/*Mse*I-CTT, *Eco*RI-TG-FAM (blue fluorescence)/*Mse*I-CAA, *Eco*RI-TG-FAM/*MSe*I-CAC, *Eco*RI-TG-FAM/*Mse*I-CTG, *Eco*RI-TG-FAM/*Mse*I-CTT, *Eco*RI-TT-NED (yellow fluorescence)/*Mse*I-CAA, *Eco*RI-TT-NED/*MSe*I-CAC, *Eco*RI-TT-NED/*Mse*I-CTG, *Eco*RI-TT-NED/*Mse*I-CTT. PCR amplifications were done in a total volume of 10 μL containing 1.5 μL of DNA from the pre-selective amplification step, 0.5 μL of *Mse*I 5 un primer, 0.5 μL *Eco*RI 1 un primer, 1 μL of 10x buffer, 0.3 μL of MgCl_2_ 50 mM, 0.1 μL of dNTP 10 mM, 0.05 of μL Platinum *Taq* DNA polymerase (Invitrogen/Thermo Fisher Scientific, Inc.) (5u/μL), and 6.05 μL of water. Cycling conditions followed the instructions in the AFLP Plant Mapping Protocol.

The samples obtained with the *Mse*I-CAA and *Mse*I-CAC extensions were analyzed on an ABI PRISM 310 Genetic Analyzer (Applied Biosystems) at the Laboratory of Plant Molecular Genetics (Embrapa Uva e Vinho, Bento Gonçalves, RS, Brazil) using 12 μL of formamide, 0.3 μL of GeneScan Rox-500 and 1 μL of undiluted PCR product. Samples with *Mse*I-CTG and *Mse*I-CTT extensions were analyzed on an ABI3730 Genetic Analyzer at the Human Genome and Stem Cell Research Center (Universidade de São Paulo, São Paulo, SP, Brazil) using 8.925 μL of formamide, 0.3 μL of GeneScan Rox-500 and 1μL of undiluted PCR product. A signal-detection threshold of 100 RFU was applied, and markers between 50 and 500 bp were selected for analysis. GeneMapper v.5 was used to generate a presence/absence (1/0) matrix for each of the markers. Table 1 shows the total number of markers obtained from each combination of primers.

**Table 1:** Polymorphic markers obtained with the *Eco*RI and *Mse*I primers used in this study.

Extension *Eco*RI	Extension *Mse*I				Total
	CAA	CAC	CTG	CTT	
TA	372	346	440	319	1477
TT	86	55	193	138	472
TG	242	65	406	352	1065
Total	700	466	1039	809	3014

We analyzed three microsatellite loci: Hel-01, Hel-08 and Hel-13 (Flanagan *et al.*, 2002), all labeled with FAM (blue fluorescence). Microsatellites, or SSR markers, are co-dominant and highly polymorphic (Hall *et al.*, 2010), though more laborious to obtain than AFLPs (Erickson *et al.*, 2004). Details of the primers used are shown in Table 2. PCR amplification was performed in a total volume of 10 μL containing 1 μL of DNA (50 ng/μL), 0.3 μL of MgCl_2_ 50 mM, 0.1 μL of dNTP 10mM, 1 μL of buffer 10x, 0.1 μL of primer forward+reverse (10 μM), 0.05 μL of Platinum *Taq* DNA polymerase (5u/μL) and 7.45 μL of water. PCR products were analyzed on an ABI3730 Genetic Analyzer at Macrogen Inc (Seoul, South Korea). Results were analyzed using GeneMapper 5. A presence/absence matrix, similar to that obtained for the AFLPs, was created for each allele. In addition, since microsatellites are co-dominant, a genotype table was also generated for each locus.

**Table 2:** Microsatellite primers and PCR amplification conditions used in this work.

*Locus*	Primer name	Chromosome	Primer sequence	Repetition	Allele number	Annealing temperature (ºC)
Hel-01	B6R	Z	5’-TCGTAGATATCCATTACTCTGGTCTG-3’	(GA)_21_	18	54
	B6F		5’-AGGGCGTCGTTAGTTTGTGT-3’			
Hel-08	A2F	12	5’-ACATCTCAGAACTGGTCGGC-3’	(CA)_14_	8	55
	A2R		5’-CTCGATCAGCCGGTGATTAT-3’			
Hel-13	CA13F	2	5’-ATTTCATAGTAACGCCCTCC-3’	(CA)_13_	8	52
	CA13R		5’-TGACTTATCGCTAAGGTCAA-3’			

### Statistical analysis

To identify AFLP and SSR markers associated with the phenotypes under consideration, we performed a chi-square (χ^2^) single marker association test on the presence/absence matrix for each AFLP and SSR marker, using Microsoft Excel 2007 and PASW Statistics for Windows, version 18.0 (SPSS Inc., Chicago, USA). Yates’ correction was applied to the chi-square tests on the 2x2 contingency tables for AFLP markers and the cannibalism/non-cannibalism phenotype (experimental design 1). Markers found to be statistically associated with the phenotypes were subjected to a logistic regression analysis with three “null” markers as covariates. Null markers are not in linkage disequilibrium with the gene being tested for association (Setakis *et al.*, 2006) and are, thus, assumed to have a neutral effect on the phenotype of interest (Abdurakhmonov and Abdukarimov, 2008). They were included in the regression analysis to reduce the effect of population structure (Balding, 2006). Logistic regression was also performed using PASW Statistics for Windows, version 18.0.

In addition, a genotype association test was performed for all microsatellite markers, again through the application of a χ^2^ test. Finally, a further χ^2^ test was conducted on the full spectrum of alleles at each microsatellite locus. This analysis is different from the analysis of individual alleles described above, as it considers all alleles at a locus together.

## Results

General information about butterfly families genotyped as part of this work is shown in Tables 3 and 4. The Yates-corrected χ^2^ test identified associations between 72 of 3014 AFLP markers and the cannibalism/non-cannibalism phenotype from experimental design 1 (see Table S1 for details on these markers). For 20 markers, the association remained statistically significant after logistic regression (Table 5).

**Table 3:** Genotyped families, for experiment 1 (two behavioral phenotypes: cannibals (C) and non-cannibals (NC)). The table shows the identification of each family, the behavior displayed by parents, the inbreeding coefficient of the offspring (F), the absolute frequency of the behavior observed by the offspring, the total number of offspring that was generated, the number of genotyped offsping, and the sex if genotyped parents.

Family	Parent’s Behavior		F	Offspring’s Behavior		Total number of offspring	Number of genotyped offspring	Sex of genotyped parents
	Mother	Father		C	NC			
1	C	C	0.25	2	13	15	2	M
2	NC	NC	0.25	6	2	8	2	M
3	NC	C	0	17	12	29	7	M
4	C	NC*	0	5	4	9	6	F, M*
5	NC	C	0	16	3	19	8	F, M
6	C	C	0	6	4	10	8	F
7	C	NC*	0	12	2	14	10	F, M*
8	C	C	0.25	2	12	14	12	M
9	NC	C	0.25	6	14	20	10	M
10	C	NC	0	20	18	38	9	F, M
11	NC	C	0	13	21	34	10	F, M
12	C	C	0	4	2	6	5	M
13	NC	NC	0.25	4	14	18	8	F
14	NC	C	0	7	6	13	7	-
15	NC	NC	0.25	4	10	14	4	F
16	C	NC	0	17	14	31	6	-
17	NC	NC	0	13	9	22	6	-
TOTAL				154	160	314	120	18

* Single individual. M = Male; F = Female.

**Table 4:** Genotyped families, from experimental design 2 (separation of four behavioral phenotypes: cannibal recognizing siblings (CR), cannibal that does not recognize siblings (CNR), super cannibal (SC) and super non-cannibal (SNC)). Abbreviations are the same as in Table 3.

Family	Parent’s behavior		F	Offsprig’s behavior				Total number of offspring	Number of genotyped offspring	Sex of genotyped parents
	Mother	Father		CR	CNR	SC	SNC			
1	CR	CNR	0	7	6	7	3	23	16	M
2	CNR	SNC*	0	8	5	1	5	19	9	F, M*
3	CNR	SNC*	0	15	4	3	15	37	24	F, M*
4	SC	SC	0	2	4	2	3	11	7	F, M
Total				32	19	13	26	90	56	6

* Single individual. M = Male; F = Female.

**Table 5:** AFLP markers that showed significant *P* values in χ^2^ tests and logistic regression between the presence (1) and absence of allele (0) and the cannibal and non-cannibal phenotypes. The extensions used (to *Eco*RI and *Mse*I) to obtain the fragment, and also the fragment size (bp) are also indicated. In columns “Non-cannibal” and “Cannibal” are shown the absolute frequencies observed for the presence and absence of the band for each behavioral phenotype. The Mendelian segregation of markers was tested when possible, and are represented in bold in column Marker.

Marker	Extensions		bp	Non-cannibal		Cannibal		χ^2^_Yates_	*P*_*x*^2^_	*P*_*Logistic Regression*_
	*Eco*RI-	*Mse*I-		0	1	0	1			
**49**	TG	CTG	90	50	15	35	27	5.119	0.024^a^	0.048
55	TG	CTG	96	57	8	62	0	6.192	0.013^b^	0.008
97	TG	CTG	130	58	7	62	0	5.150	0.023^b^	0.027
**206**	TG	CTG	219	31	34	49	13	12.059	0.001^b^	0.003
**275**	TG	CTG	280	65	0	56	6	4.627	0.031^a^	0.034
**276**	TG	CTG	281	62	3	49	13	6.292	0.012^a^	0.040
**447**	TA	CTG	83	56	9	42	21	5.728	0.017^a^	0.026
**531**	TA	CTG	156	57	8	63	0	6.304	0.012^b^	0.016
**983**	TT	CTG	205	47	10	51	1	5.693	0.017^b^	0.050
**1092**	TG	CTT	100	54	11	62	1	7.143	0.008^b^	0.040
**1098**	TG	CTT	103	27	38	42	21	7.150	0.007^b^	0.029
**1120**	TG	CTT	124	15	50	32	31	9.418	0.002^b^	0.026
**1127**	TG	CTT	130	59	6	43	20	8.677	0.003^a^	0.008
**1295**	TG	CTT	267	56	9	36	27	11.923	0.001^a^	0.000
**1306**	TG	CTT	277	64	1	53	10	6.643	0.010^a^	0.020
**1358**	TG	CTT	344	65	0	56	7	5.642	0.018^a^	0.017
**1484**	TA	CTT	127	61	4	49	14	5.570	0.018^a^	0.031
**1563**	TA	CTT	205	64	1	49	14	11.306	0.001^a^	0.000
**2018**	TA	CAA	196	68	0	64	6	4.207	0.040^a^	0.033
**2314**	TG	CAA	232	65	3	57	13	5.436	0.020^a^	0.032

a presence of marker most common in cannibals.

b presence of marker most common in non-cannibals.

In the single-marker test of the phenotypes of experimental design 2 (cannibal that recognizes siblings, CR; cannibal that does not recognize siblings, CNR; “super cannibal”, SC; and “super non-cannibal”, SNC), a statistically significant association was found for 73 AFLPs (see Table S2 for details on these markers). For 11 markers, the association remained statistically significant after logistic regression (Table 6).

**Table 6:** AFLP markers that showed significant *P* values in χ^2^ tests and logistic regression, for association between the presence (1) and absence (0) of allele and phenotypes “cannibals which recognize siblings” (CR), “cannibals which does not recognize siblings” (CNR), “super cannibal” (SC) and “super non-cannibal” (SNC). The extensions used are indicated (*Eco*RI and *Mse*I) to obtain fragment, and also the fragment size (bp). In columns “CR”, “CNR”, “SC” and “SNC” the absolute frequencies are shown for the presence/absence of the band for each behavioral phenotype. The Mendelian segregation of markers was tested when possible, and is represented in bold in column Marker.

Marker	Extensions		bp	CR		CNR		SC		SNC		χ^2^	*P*_*x*^2^_	*P*_*Logistic Regression*_
	*Eco*RI-	*Mse*I-		0	1	0	1	0	1	0	1			
**473**	TA	CTG	104	14	5	10	5	8	3	3	14	14.5	0.002^d^	0.025
**942**	TT	CTG	148	17	2	7	8	6	5	16	1	14.0	0.003^b^ ^c^	0.035
1122	TG	CTT	125	19	0	9	6	10	1	17	0	17.0	0.001^b^ ^c^	0.020
**1171**	TG	CTT	165	14	0	13	2	11	0	7	10	14.1	0.003^d^	0.024
**1185**	TG	CTT	176	19	5	13	2	11	0	8	9	21.1	< 0.001^d^	0.002
**1466**	TA	CTT	114	5	14	5	10	10	1	8	9	13.0	0.005^a^ ^b^ ^d^	0.040
2133	TA	CAA	82	11	8	9	6	11	0	17	0	15.0	0.002^a^ ^b^	0.009
**2151**	TA	CAA	96	2	17	10	5	3	8	2	15	16.4	0.001^a^ ^d^	0.021
**2168**	TA	CAA	108	19	0	11	4	8	3	17	0	10.9	0.012^b^ ^c^	0.026
**2215**	TA	CAA	146	11	8	8	7	7	4	17	0	10.7	0.014^a^ ^b^ ^c^	0.040
**2493**	TT	CAA	78	13	6	8	7	7	4	2	15	13.3	0.004^d^	0.031

a presence of marker most common in CR.

b presence of marker most common in CNR.

c presence of marker most common in SC.

d presence of marker most common in SNC.

The results of association analyses between the microsatellite markers (Hel-01, Hel-08 and Hel-13) and the phenotypes of experimental design 1 (C and NC) are shown in Table 7. For Hel-13, a significant association was found in the test considering all alleles together (*P* = 0.004; 7 df). Alleles 229 bp, 241 bp and 255 bp were more common in non-cannibals, and alleles 233 bp, 235 bp and 237 bp were more common in cannibals. The *P* value for Hel-08 was near significance (*P* = 0.051; 7 df). Allele 283 bp was more common in non-cannibals, and allele 296 bp was more common in cannibals. Genotype association analysis identified an association with the Hel-08 locus (*P* = 0.017; 18 df). Genotypes 273pb / 273pb and 273pb / 279pb were more common in cannibals, and genotypes 281pb / 283pb and 273pb / 283pb were more common in non-cannibals.

**Table 7:** Microsatellite markers that resulted in a statistically significant association between the presence/absence of the mark and behavioral phenotype. The name of the locus, allele size (bp), the absolute frequencies of presence/absence of alleles for each behavioral phenotype, the value of χ^2^ with Yates correction, the corresponding *P* value, and the value of *P* resulting logistic regression are all shown.

Locus	bp	Non-cannibal		Cannibal		χ^2^_Yates_	*P*_*x*^2^_	*P*_*Logistic regression*_
		0	1	0	1			
Hel-08	283	31	34	48	19	6.91	0.009	0.034
Hel-13	229	45	21	27	38	8.34	0.002	0.014
Hel-13	233	50	15	37	29	4.49	0.019	0.038

The analysis of the microsatellite markers and the phenotypes of experimental design 2 (CR, CNR, SC, and SNC) identified a single significant association based on the presence or absence of each individual allele, for the 403 bp allele at the Hel-01 locus (*P* = 0.027 in the χ^2^ test). However, statistical significance was not maintained after logistic regression (*P* = 0.106). No associations were found for these phenotypes in the joint analysis of all alleles and in the genotypic analyses.

After logistic regression, none of the markers showed a significant association with the phenotypes of both experimental designs. However, based on the χ^2^ test, five markers (AFLPs 78, 146, 315, 1484 and 1563) were found to be associated with the phenotypes tested in both experimental designs. Details of these markers can be found in Tables S1 and S2.

## Discussion

Our preliminary study identified a number of associations between molecular markers and phenotypes for both experimental designs employed here. For experimental design 1, which distinguished between cannibal and non-cannibal phenotypes, associations with the non-cannibal phenotype were found for AFLPs 206, 1098, and 1120, and for the SSRs Hel-08 and Hel-13, as well as for the cannibal phenotype (49, 447, 1127, 1295). For experimental design 2, which distinguished between four behavioral phenotypes (cannibal that recognizes siblings, cannibal that does not recognize siblings, super cannibal and super non-cannibal), a number of observations were made. Certain markers, such as AFLP 473, were more common in super non-cannibals (SNC); others, including AFLP 2151, were more common in cannibals that recognize siblings and in super non-cannibals, both being phenotypes related to kin recognition. On the other hand, there were markers, such as AFLPs 1122 and 2168, that were absent in these phenotypes.

The striking frequency of “kin recognition” behavioral phenotypes observed in the offspring of family 3, which was obtained as part of experiment 2 (Table 4), is particularly noteworthy. The family produced a total of 37 offspring. Of these, 40.5% cannibalized unrelated eggs only, 40.5% did not cannibalize any eggs, 11% were cannibals that did not recognize siblings, and 8% were super-cannibals. These figures mean that 81% of the offspring from this family showed kin recognition behavior. This high prevalence of kin recognition within a family is in accordance with our previous report, in which we presented evidence for the heritability of kin recognition (De Nardin *et al.*, 2017). Another possibility, which cannot be entirely discarded, is that this unusual spectrum of phenotypes is due to the effect of genes associated with cannibalism itself, rather than kin recognition.

Hamilton (1964a,b) showed how altruism could evolve if ‘genes for altruism’ had the effect of increasing the fitness of relatives, even in the case of costs to the altruist. Thompson *et al.* (2013) formulated a set of testable hypotheses describing the evolution, expression and features expected for such genes for altruism. Genes underlying altruism: (i) should satisfy Hamilton’s Rule *rb > c*, where *r* is the genetic relationship between the altruist and recipient, *b* is the benefit for the recipient, and *c* the cost to the altruist; (ii) should be environmentally sensitive; (iii) should increase in number and complexity with increasing social-behavioral organization; (iv) should coevolve with or depend on the previous evolution of genes for kin recognition; (v) may reside in regions of low recombination, to show co-expression and modular genetic architecture; (vi) should be at least partially additive, and (vi) should exhibit strong pleiotropy. From our own experience in the field and laboratory (De Nardin J, 2012, MSc thesis, Universidade Federal do Rio Grande do Sul, Porto Alegre, RS, Brazil; De Nardin and Araújo, submitted), the avoidance of sibling egg-cannibalism by caterpillars of *H. erato phyllis* is compatible with Hamilton’s rule. We furthermore observed, both in the field and experimentally, an environmental dependency of cannibalistic behavior (Huff, De Nardin and Araújo, unpublished results). If there are genes for cannibalism, condition (ii) above could be fulfilled.

Association analyses have been used as a tool for the identification of population-wide polymorphisms associated with particular phenotypes (Parker TB, 2007, Doctoral thesis. Oregon State University, USA). These associations arise due to the joint transmission of phenotypes and genotypes over many generations. Association analysis does not model these transmissions directly, although linkage analysis does. Relationships between individuals are central to linkage analysis; in association analysis, these relationships are usually distant or unknown, and where present, close relationships are a complicating factor (Astle and Balding, 2009). Population structure, or stratification, can lead to spurious associations (association without linkage) between a candidate marker and the phenotype. Several methods have been developed to reduce such spurious associations (Pritchard *et al.*, 2000; Wang *et al.*, 2005; Yu *et al.*, 2005; Zhu *et al.*, 2008; Zhang *et al.*, 2009; Thornton *et al.*, 2014). Here, this was achieved by using null markers as covariates in the logistic regression analysis (Wang *et al.*, 2005; Setakis *et al.*, 2006).

Because our analyses included families, genetic structure was an issue. However, our experiments could not reasonably have been performed in a random population sample. The kin recognition phenotypes studied here (cannibal/non-cannibal) could not have been determined other than through a laboratory-based behavioral test. Therefore, we have chosen to perform various kinds of crosses between parents with different behavioral phenotypes, and from different populations. We have included inbred crosses in our approach, which we know occur in nature (Di Mare and Araújo, 1986). Moreover, all behavioral phenotypes, both from experiment 1 and experiment 2, were present in the offspring of all families. That is, the subgroups are phenotypically similar in terms of the characteristics here analyzed, despite possible genetic differentiation. Furthermore, we corrected spurious associations by logistic regression using neutral, unlinked markers, a common practice in genetic association studies. Of the 72 associations initially identified by the χ^2^ test in experiment 1, only 20 remained statistically significant after logistic regression with null markers. Of the 73 associations initially found in experiment 2, 11 remained significant. Setakis *et al.* (2006) conducted a simulation study to compare the merits of different methods that use null (unlinked) markers, to protect against critical substructures in genetic association studies. One of the most important findings from their study was that simple statistical procedures, based on logistic regression, performed well in all scenarios considered. Methods based on logistic regression do not require an estimate of the number of underlying subpopulations; in fact, they dispense entirely with the notion of subpopulation. One possible explanation for their effectiveness is that each null marker included in the regression absorbs part of the effect of population stratification, but because this effect is shared across many markers, none of the markers is individually significant. Wang *et al.* (2005) showed that it is possible to control for population structure within a logistic regression model by including the genotype of a single marker that is informative about ancestry among the covariates.

To detect a QTL using single-marker tests is a simple procedure that can be performed with any statistical analysis software, and which has the potential to identify a significant number of markers (Doerge, 2002). However, some issues need to be considered in the statistical analysis of the results. The first is sample size. A large sample size provides more opportunities for the observation of recombination events, allowing to estimate parameters with high accuracy and, therefore, results in a greater ability to detect QTLs (Doerge, 2002). The size of the sample used here was not large. This was in particular the case for experiment 2, which may explain the lower number of associations found for that design. Another statistical problem associated with the use of small sample sizes is that they exaggerate the effect of a QTL on the phenotype in what is called the Beavis effect (Beavis, 1994; Erickson *et al.*, 2004). Furthermore, single-marker analysis can only detect QTLs with a relatively large influence on the trait of interest. The current study design is likely to have a relatively modest power to identify QTLs. A further problem is introduced by the investigation of many markers using independent statistical tests, or multiple testing. This problem is related to the level of statistical significance which is established by the investigator and can lead to the detection of false positives. Typically, researchers tolerate the incorrect detection of a QTL in 5% of the cases (Doerge, 2002). Associations that appear purely by chance are called “false positives”, or type I errors. Type I errors can be minimized by establishing more stringent criteria for statistical significance, e.g. by applying a Bonferroni correction. However, as type I errors diminish, QTLs with small effect sizes are increasingly unlikely to be detected, thus increasing the probability of type II errors, or false negatives (Grisel, 2000).

Another limitation of single-marker strategies is that they fail to provide the frequency of recombination between the marker and the QTL, and thus the precise location of the QTL. This is because the effect of the QTL and its location are conflated and cannot be estimated separately (Doerge, 2002). The work presented here thus only represents a preliminary search for possible associations between genotype and phenotype. It does not aim at the exact mapping of QTLs, and to do so would require a single segregating population originating from a cross between individuals of contrasting phenotypes, and a fairly large number of offspring of at least 50 to 250 individuals would be needed to allow preliminary mapping (Collard *et al.*, 2005), which was not possible here.

The ability to detect a QTL depends on the magnitude of its effect on the trait of interest, the size of the segregating population evaluated, the frequency of recombination between the marker and the QTL, as well as the heritability of the trait. The larger the effect size and the size of the population, the greater the heritability, and the smaller the distance between the QTL and the marker, the easier it becomes to detect the QTL (Ferreira and Gratapaglia, 1995). We have previously estimated the heritability of the non-cannibalism phenotype to lie around 20% (De Nardin *et al.*, 2017); this relatively low value makes it difficult to detect the underlying QTLs.

QTLs affecting various quantitative characteristics, such as life span, starvation, reproductive success, number of sensory bristles, sex comb teeth and ovarioles, flight velocity and metabolic traits, courtship song, locomotor behavior and male mating and aggressive behavior have been mapped in *Drosophila melanogaster* (reviewed by Edwards and Mackay, 2009). Studies in honeybees (*Apis mellifera)* have identified QTLs that influence the expression of foraging and defensive behavior in colonies, specific individual behavior, the tendency of individuals perform guard and stinging behavior (reviewed by Arechavaleta-Velasco *et al.*, 2003), as well as hygienic behavior (Lapidge *et al.*, 2002). Caillaud and Via (2012) studied QTLs related to feeding behavior in the pea aphid *Acyrthosiphon pisum*. It should be emphasized that all of these studies deal with traits that can be measured on a continuous scale, while the phenotypes studied here, although they are also quantitative traits, are observed in a nonlinear way.

Linkage maps are available for *Heliconius melpomene* (Jiggins *et al.*, 2005) and *Heliconius erato* (Tobler *et al.*, 2005; Kapan *et al.*, 2006). This has enabled the estimation of the sizes of these species’ genomes. Both comprise 21 chromosomes, with a total of 1616 cM or 292 Mb for *H. melpomene* (Jiggins *et al.*, 2005) and 2400 cM or 395 Mb for *H. erato* (Tobler *et al.*, 2005). These maps were obtained by crossing individuals with contrasting phenotypes for a trait of interest (either within or even between species), and analyzing a large number of offspring (> 70 individuals) from each cross. We could not implement this approach for several reasons: (i) our phenotyping strategy depended on testing for cannibalism, which used three eggs at a time, meaning that not all offspring generated by a female butterfly reached adulthood; (ii) numerous tests had to be cancelled, either because cannibalism could not be detected unambiguously (due to bite marks on an egg not being clearly visible), because the stipulated test duration was exceeded, or because the eggs dried up during the test; (iii) many females died prematurely; (iv) many females laid only few eggs. It is important to note that all linkage maps are unique products of the population (derived from two specific parents) and the types of markers used (Collard *et al.*, 2005). Therefore, even if we had performed many crosses between individuals of different populations, we would not be able to tell with certainty that all associations found here would be observed in other individuals from other populations.

We are aware of the potential problems inherent in our methodology, as well as of the limitations of this kind of preliminary study. Specifically, QTL analysis by a single marker does not allow to establish the location of a particular locus, thus preventing a linkage map. Nevertheless, our data indicate the likely presence of associations between AFLP and SSR markers and the behavioral phenotypes studied. We tested around 3,000 markers, an average of 7.6 markers per Mb. The sampling of genetic variability however was sufficiently broad to ensure the reliability of the data and conclusions. Nevertheless, additional studies will be necessary to validate the associations found here, and to construct a linkage map of the markers used here, with the ultimate aim of identifying and mapping the main genetic factors involved in the control of the assessed phenotypes and measuring the magnitude of their effect. Likewise, a larger number of markers will have to be tested in regions harboring association signals to define more accurately the location of the loci of interest.

## Supplementary material

The following online material is available for this article:

Table S1 - Significant AFLP markers: association of allele presence/absence with cannibalism phenotypes.

Table S2 - Significant AFLP markers: association of allele presence/absence with cannibalism/kin recognition phenotypes.

## References

[B1] Abdurakhmonov IY, Abdukarimov A (2008). Application of association mapping to understanding the genetic
diversity of plant germplasm resources. Int J Plant Genomics.

[B2] Arechavaleta-Velasco ME, Hunt GJ, Emore C (2003). Quantitative trait *loci* that influence the
expression of guarding and stinging behaviors of individual honey
bees. Behav Genet.

[B3] Astle W, Balding DJ (2009). Population structure and cryptic relatedness in genetic
association studies. Stat Sci.

[B4] Balding DJ (2006). A tutorial on statistical methods for population association
studies. Nat Rev Genet.

[B5] Beavis WD, Wilkinson DB (1994). The power and deceit of QTL experiments: Lessons
from comparative QTL studies. Proceedings of the Forty-Ninth Annual Corn and Sorghum Industry Research
Conference.

[B6] Breed MD (2014). Kin and nestmate recognition: The influence of W. D. Hamilton on 50 years of research. Anim Behav.

[B7] Brown KS (1981). The biology of *Heliconius* and related
genera. Annu Rev Entomol.

[B8] Bush WS, Moore JH (2012). Chapter 11: Genome-wide association studies. PLoS Comp Biol.

[B9] Caillaud MC, Via S (2012). Quantitative genetics of feeding behavior in two ecological races
of the pea aphid, *Acyrthosiphon pisum*. Heredity.

[B10] Collard BCY, Jahufer MZZ, Brouwer JB, Pang ECK (2005). An introduction to markers, quantitative trait
*loci* (QTL) mapping and marker-assisted selection for
crop improvement: The basic concepts. Euphytica.

[B11] De Nardin J, Araújo AM (2011). Kin recognition in immatures of *Heliconius erato
phyllis* (Lepidoptera; Nymphalidae). J Ethol.

[B12] De Nardin J, Da Silva L, Araújo AM (2017). Kin recognition in a butterfly: inferences about its
heritability. Ethol Ecol Evol.

[B13] Di Mare RA, Araújo AM (1986). A first survey of inbreeding effects in *Heliconius erato
phyllis* (Lepidoptera; Nymphalidae). Rev Bras Genet.

[B14] Doerge RJ (2002). Mapping and analysis of quantitative trait *loci*
in experimental populations. Nat Rev Genet.

[B15] Edwards AC, Mackay TFC (2009). Quantitative trait *loci* for aggressive behavior
in *Drosophila melanogaster*. Genetics.

[B16] Erickson DL, Fenster CB, StenØien HK, Price D (2004). Quantitative trait *locus* analyses and the study
of evolutionary process. Mol Ecol.

[B17] Falconer DS, Mackay TFC (1996). Introduction to Quantitative Genetics.

[B18] Ferreira ME, Gratapaglia D (1995). Introdução ao uso de marcadores RAPD e RFLP em análise genética.

[B19] Flanagan NS, Blum MJ, Davison A, Alamo M, Albarrán R, Faulhaber K, Peterson E, McMillan WO (2002). Characterization of microsatellite loci in neotropical
*Heliconius* butterflies. Mol Ecol Notes.

[B20] Ford EB (1964). Ecological Genetics.

[B21] Ford EB (1975). Ecological Genetics.

[B22] Grisel JE (2000). Quantitative trait *locus*
analysis. Alcohol Res Health.

[B23] Hall D, Tegström C, Ingvarsson PK (2010). Using association mapping to dissect the genetic basis of complex
traits in plants. Brief Funct Genomics.

[B24] Hamilton WD (1964a). Genetical evolution of social behavior. I. J Theor Biol.

[B25] Hamilton WD (1964b). Genetical evolution of social behavior. II. J Theor Biol.

[B26] Jiggins CD, Mavarez J, Beltrán M, McMillan WO, Johnston JS, Bermingham E (2005). A genetic linkage map of the mimetic butterfly *Heliconius
melpomene*. Genetics.

[B27] Joron M, Frezal L, Jones RT, Chamberlain NL, Lee SF, Haag CR, Whibley A, Becuwe M, Baxter SW, Ferguson L (2011). Chromosomal rearrangements maintain a polymorphic supergene
controlling butterfly mimicry. Nature.

[B28] Kapan DD, Flanagan NS, Tobler A, Papa R, Reed RD, Gonzalez JA, Restrepo MR, Martinez L, Maldonado K, Ritschoff C (2006). Localization of Müllerian mimicry genes on a dense linkage map of
*Heliconius erato*. Genetics.

[B29] Kettlewell HBD (1955). Selection experiments on industrial melanism in the
Lepidoptera. Heredity.

[B30] Lapidge KL, Oldroyd BP, Spivak M (2002). Seven suggestive quantitative trait *loci*
influence hygienic behavior of honey bees. Naturwissenschaften.

[B31] Lynch M, Walsh B (1998). Genetics and Analysis of Quantitative Traits.

[B32] Mega NO, Revers LF (2011). Developing a rapid, efficient and low cost method for rapid DNA
extraction from arthropods. Cienc Rural.

[B33] Merrill RM, Dasmahapatra KK, Davey JW, Dell’Aglio DD, Hanly JJ, Huber B, Jiggins CD, Joron M, Kozak KM, Llaurens V (2015). The diversification of *Heliconius* butterflies:
What have we learned in 150 years?. J Evol Biol.

[B34] Olson JM, Witte JS, Elston RC (1999). Tutorial in biostatistics genetic mapping of complex
traits. Stat Med.

[B35] Pritchard JK, Stephens M, Donnelly P (2000). Inference of population structure using multilocus genotype
data. Genetics.

[B36] Ramos RR, Freitas AVL (1999). Population biology and wing color variation in *Heliconius
erato phyllis* (Nymphalidae). J Lepid Soc.

[B37] Roff DA, Tucker J, Stirling G, Fairbairn DJ (1999). The evolution of threshold traits: Effects of selection on
fecundity and correlated response in wing dimorphism in the sand
cricket. J Evol Biol.

[B38] Setakis E, Stirnadel H, Balding DJ (2006). Logistic regression protects against population structure in
genetic association studies. Genome Res.

[B39] Thompson GJ, Hurd PL, Crespi BJ (2013). Genes underlying altruism. Biol Lett.

[B40] Thornton T, Conomos MP, Sverdlov S, Blue EM, Cheung CYK, Glazner CG, Lewis SM, Wijsman EM (2014). Estimating and adjusting for ancestry admixture in statistical
methods for relatedness inference, heritability estimation, and association
testing. BMC Proc.

[B41] Tobler A, Kapan D, Flanagan NS, Gonzalez C, Peterson E, Jiggins CD, Johntson JS, Heckel DG, McMillan WO (2005). First-generation linkage map of the warningly colored butterfly
*Heliconius erato*. Heredity.

[B42] Wang Y, Localio R, Rebbeck TR (2005). Bias correction with a single null marker for population
stratification in candidate gene association studies. Hum Hered.

[B43] Wu R, Ma CX, Casella G (2002). Joint linkage and linkage disequilibrium mapping of quantitative
trait *loci* in natural populations. Genetics.

[B44] Yu J, Pressoir G, Briggs WH, Bi IV, Yamasaki M, Doebley JF, McMullen MD, Gaut BS, Nielsen DM, Holland JB (2005). A unified mixed-model method for association mapping that
accounts for multiple levels of relatedness. Nat Genet.

[B45] Zhang L, Li J, Pei YF, Liu Y, Deng HW (2009). Tests of association for quantitative traits in nuclear families
using principal components to correct for population
stratification. Ann Hum Genet.

[B46] Zhu X, Li S, Cooper RS, Elston RC (2008). A unified association analysis approach for family and unrelated
samples correcting for stratification. Am J Hum Genet.

