# Supplementary material for: Association between molecular markers and behavioral phenotypes in the immatures of a butterfly
Source: Genet Mol Biol. 2018 Mar 19;41(1 Suppl 1):243–52. doi: 10.1590/1678-4685-GMB-2017-0073 (PMC5913723; doi:10.1590/1678-4685-GMB-2017-0073)
Supplement: Supplementary file 1 [file 1415-4757-GMB-41-01-2017-0073-s001.pdf]

# Supplementary Material to "Association between molecular markers and behavioral phenotypes in the immatures of a butterfly"

**Table S1** - AFLP markers that showed significant P values in $\chi^2$ tests between the presence (1) and absence of allele (0) and the cannibal and non-cannibal phenotypes. The extensions used (for *Eco*RI and *Mse*I) to obtain the fragment, and also the fragment size (bp) are also indicated. In columns "Non-cannibal" and "Cannibal" are shown the absolute frequencies observed for the presence and absence of the band for each behavioral phenotype. The Mendelian segregation of markers was tested when possible, and is represented in bold in the column Marker.

| Marker | Extensions | | bp | Non-cannibal | | Cannibal | | $\chi^2$Yates | *P* $\chi^2$ | *P* Logistic regression |
|---|---|---|---|---|---|---|---|---|---|---|
| | ***Eco*RI-** | ***Mse*I-** | | **0** | **1** | **0** | ***1*** | | | |
| 48 | TG | CTG | 89 | 50 | 15 | 57 | 5 | 4.318 | 0.038 | 0.155 |
| **49** | TG | CTG | 90 | 50 | 15 | 35 | 27 | 5.119 | 0.024 | 0.048 |
| 55 | TG | CTG | 96 | 57 | 8 | 62 | 0 | 6.192 | 0.013 | 0.008 |
| **58** | TG | CTG | 98 | 31 | 34 | 42 | 20 | 4.431 | 0.035 | 0.185 |
| **61** | TG | CTG | 101 | 46 | 19 | 56 | 6 | 6.487 | 0.006 | 0.056 |
| **65** | TG | CTG | 105 | 54 | 11 | 60 | 2 | 5.074 | 0.024 | 0.092 |
| **78** | TG | CTG | 114 | 22 | 43 | 35 | 27 | 5.673 | 0.017 | 0.377 |
| **82** | TG | CTG | 117 | 19 | 46 | 30 | 32 | 4.139 | 0.042 | 0.482 |
| 97 | TG | CTG | 130 | 58 | 7 | 62 | 0 | 5.150 | 0.023 | 0.027 |
| **113** | TG | CTG | 144 | 29 | 36 | 43 | 19 | 6.935 | 0.008 | 0.087 |
| **124** | TG | CTG | 153 | 56 | 9 | 43 | 19 | 4.279 | 0.039 | 0.125 |
| **145** | TG | CTG | 169 | 49 | 16 | 34 | 28 | 5.043 | 0.025 | 0.085 |
| **146** | TG | CTG | 170 | 26 | 39 | 37 | 25 | 4.159 | 0.041 | 0.433 |
| 154 | TG | CTG | 176 | 58 | 7 | 46 | 16 | 3.877 | 0.049 | 0.111 |
| **163** | TG | CTG | 184 | 53 | 12 | 60 | 2 | 6.037 | 0.014 | 0.102 |
| **206** | TG | CTG | 219 | 31 | 34 | 49 | 13 | 12.059 | 0.001 | 0.003 |
| **217** | TG | CTG | 227 | 37 | 28 | 48 | 14 | 5.132 | 0.023 | 0.228 |
| **226** | TG | CTG | 234 | 48 | 17 | 56 | 6 | 4.751 | 0.029 | 0.171 |
| **275** | TG | CTG | 280 | 65 | 0 | 56 | 6 | 4.627 | 0.031 | 0.034 |
| **276** | TG | CTG | 281 | 62 | 3 | 49 | 13 | 6.292 | 0.012 | 0.040 |

| **Marker** | **Extensions** | | **bp** | **Non-cannibal** | | **Cannibal** | | **$\chi^2$Yates** | ***P* $\chi^2$** | ***P* Logistic regression** |
|---|---|---|---|---|---|---|---|---|---|---|
| | ***Eco*RI-** | ***Mse*I-** | | **0** | **1** | **0** | ***1*** | | | |
| **315** | TG | CTG | 327 | 63 | 2 | 53 | 9 | 3.902 | 0.048 | 0.145 |
| **345** | TG | CTG | 360 | 59 | 6 | 62 | 0 | 4.131 | 0.042 | 0.070 |
| 447 | TA | CTG | 83 | 56 | 9 | 42 | 21 | 5.728 | 0.017 | 0.026 |
| 454 | TA | CTG | 89 | 57 | 8 | 62 | 1 | 4.104 | 0.043 | 0.173 |
| **465** | TA | CTG | 97 | 14 | 51 | 25 | 38 | 4.152 | 0.042 | 0.142 |
| **471** | TA | CTG | 102 | 46 | 19 | 55 | 8 | 4.307 | 0.038 | 0.309 |
| **531** | TA | CTG | 156 | 57 | 8 | 63 | 0 | 6.304 | 0.012 | 0.016 |
| **533** | TA | CTG | 158 | 49 | 16 | 36 | 27 | 3.989 | 0.046 | 0.090 |
| **534** | TA | CTG | 159 | 40 | 25 | 52 | 11 | 5.980 | 0.014 | 0.117 |
| 546 | TA | CTG | 168 | 47 | 18 | 56 | 7 | 4.591 | 0.032 | 0.146 |
| 599 | TA | CTG | 214 | 65 | 0 | 57 | 6 | 4.538 | 0.033 | 0.063 |
| **600** | TA | CTG | 215 | 61 | 4 | 50 | 13 | 4.635 | 0.031 | 0.094 |
| **665** | TA | CTG | 285 | 49 | 16 | 58 | 5 | 5.330 | 0.021 | 0.107 |
| **749** | TA | CTG | 374 | 51 | 14 | 60 | 3 | 6.429 | 0.011 | 0.050 |
| **884** | TT | CTG | 78 | 23 | 34 | 32 | 20 | 4.072 | 0.044 | 0.089 |
| **896** | TT | CTG | 91 | 43 | 14 | 48 | 4 | 4.456 | 0.035 | 0.084 |
| 923 | TT | CTG | 123 | 55 | 2 | 43 | 9 | 4.287 | 0.038 | 0.168 |
| **983** | TT | CTG | 205 | 47 | 10 | 51 | 1 | 5.693 | 0.017 | 0.050 |
| **1000** | TT | CTG | 223 | 37 | 20 | 21 | 31 | 5.623 | 0.018 | 0.118 |
| **1022** | TT | CTG | 276 | 56 | 1 | 44 | 8 | 4.991 | 0.025 | 0.085 |
| **1055** | TG | CTT | 67 | 53 | 12 | 60 | 3 | 4.555 | 0.033 | 0.077 |
| **1069** | TG | CTT | 79 | 36 | 29 | 48 | 15 | 5.251 | 0.022 | 0.170 |
| **1088** | TG | CTT | 96 | 22 | 43 | 35 | 28 | 5.257 | 0.022 | 0.113 |
| **1092** | TG | CTT | 100 | 54 | 11 | 62 | 1 | 7.143 | 0.008 | 0.040 |
| **1098** | TG | CTT | 103 | 27 | 38 | 42 | 21 | 7.150 | 0.007 | 0.029 |
| **1120** | TG | CTT | 124 | 15 | 50 | 32 | 31 | 9.418 | 0.002 | 0.026 |
| **1127** | TG | CTT | 130 | 59 | 6 | 43 | 20 | 8.677 | 0.003 | 0.008 |
| **1187** | TG | CTT | 177 | 64 | 1 | 55 | 8 | 4.508 | 0.034 | 0.071 |
| **1225** | TG | CTT | 205 | 28 | 37 | 41 | 22 | 5.379 | 0.020 | 0.319 |

| **Marker** | **Extensions** | | **bp** | **Non-cannibal** | | **Cannibal** | | **χ²Yates** | ***P* χ²** | ***P* Logistic regression** |
|---|---|---|---|---|---|---|---|---|---|---|
| | ***Eco*RI-** | ***Mse*I-** | | **0** | **1** | **0** | ***1*** | | | |
| **1227** | TG | CTT | 207 | 9 | 56 | 19 | 44 | 4.073 | 0.044 | 0.541 |
| **1229** | TG | CTT | 209 | 65 | 0 | 57 | 6 | 4.538 | 0.033 | 0.088 |
| **1241** | TG | CTT | 219 | 65 | 0 | 57 | 6 | 4.538 | 0.033 | 0.063 |
| **1295** | TG | CTT | 267 | 56 | 9 | 36 | 27 | 11.923 | 0.001 | 0.000 |
| **1306** | TG | CTT | 277 | 64 | 1 | 53 | 10 | 6.643 | 0.010 | 0.020 |
| **1358** | TG | CTT | 344 | 65 | 0 | 56 | 7 | 5.642 | 0.018 | 0.017 |
| **1431** | TA | CTT | 84 | 44 | 21 | 54 | 9 | 4.830 | 0.028 | 0.157 |
| 1468 | TA | CTT | 115 | 62 | 3 | 52 | 11 | 4.180 | 0.041 | 0.120 |
| **1484** | TA | CTT | 127 | 61 | 4 | 49 | 14 | 5.570 | 0.018 | 0.031 |
| **1536** | TA | CTT | 177 | 41 | 24 | 52 | 11 | 5.160 | 0.023 | 0.155 |
| 1540 | TA | CTT | 181 | 55 | 10 | 61 | 2 | 4.269 | 0.039 | 0.108 |
| **1563** | TA | CTT | 205 | 64 | 1 | 49 | 14 | 11.306 | 0.001 | 0.000 |
| **1582** | TA | CTT | 223 | 65 | 0 | 57 | 6 | 4.538 | 0.033 | 0.064 |
| 1827 | TT | CTT | 220 | 53 | 4 | 39 | 13 | 5.384 | 0.020 | 0.085 |
| 1856 | TG | CAA | 56 | 60 | 8 | 69 | 1 | 4.468 | 0.035 | 0.159 |
| **2008** | TG | CAA | 180 | 62 | 6 | 70 | 0 | 4.510 | 0.034 | 0.071 |
| **2018** | TA | CAA | 196 | 68 | 0 | 64 | 6 | 4.207 | 0.040 | 0.033 |
| **2118** | TA | CAA | 72 | 42 | 26 | 29 | 41 | 4.926 | 0.026 | 0.170 |
| **2119** | TA | CAA | 73 | 47 | 21 | 64 | 6 | 9.539 | 0.002 | 0.098 |
| **2128** | TG | CAA | 79 | 55 | 13 | 45 | 25 | 3.966 | 0.046 | 0.527 |
| **2177** | TG | CAA | 115 | 51 | 17 | 38 | 32 | 5.590 | 0.018 | 0.228 |
| **2314** | TG | CAA | 232 | 65 | 3 | 57 | 13 | 5.436 | 0.020 | 0.032 |
| 2491 | TT | CAA | 77 | 54 | 8 | 62 | 1 | 4.415 | 0.036 | 0.056 |
