# Supplementary material for: Association between molecular markers and behavioral phenotypes in the immatures of a butterfly
Source: Genet Mol Biol. 2018 Mar 19;41(1 Suppl 1):243–52. doi: 10.1590/1678-4685-GMB-2017-0073 (PMC5913723; doi:10.1590/1678-4685-GMB-2017-0073)
Supplement: Supplementary file 2 [file 1415-4757-GMB-41-01-2017-0073-s002.pdf]

Supplementary Material to "Association between molecular markers and behavioral phenotypes in the immatures of a butterfly"

Table S2 - AFLP markers that showed significant P values in χ^2 tests, for association between the presence (1) and absence (0) of allele and phenotypes "cannibals which recognize siblings" (CR), "cannibals which does not recognize siblings" (CNR), "super cannibal" (SC) and "super non-cannibal" (SNC). The extensions used are indicated (*Eco*RI and *Mse*I) to obtain fragment, and also the fragment size (bp). In columns "CR", "CNR", "SC" and "SNC" the absolute frequencies are shown for the presence/absence of the band for each behavioral phenotype. The Mendelian segregation of markers was tested when possible, and is represented in bold in the column Marker.

**Marker**	**Extensions**		**bp**	**CR**		**CNR**		**SC**		**SNC**		χ^2	***P*** χ^2	***P Logistic Regression***
	***Eco*RI-**	***Mse*I-**		**0**	**1**	**0**	**1**	**0**	**1**	**0**	**1**			
**4**	TG	CTG	53	19	0	12	3	11	0	17	0	9.878	0.020	0.275
**17**	TG	CTG	64	2	17	8	7	3	8	3	14	8.857	0.031	0.205
18	TG	CTG	65	15	4	11	4	5	6	16	1	8.786	0.032	0.155
**31**	TG	CTG	76	19	0	11	4	11	0	16	1	9.742	0.021	0.132
32	TG	CTG	77	19	0	15	0	9	2	17	2	9.582	0.022	0.369
**78**	TG	CTG	114	5	10	3	16	5	6	0	17	10.182	0.017	0.072
**118**	TG	CTG	148	19	0	11	4	10	1	17	0	10.174	0.017	0.186
**146**	TG	CTG	170	7	12	4	11	6	5	13	4	9.383	0.025	0.206
**168**	TG	CTG	187	11	8	7	8	10	1	15	2	10.063	0.018	0.218
**175**	TG	CTG	194	14	5	5	10	9	2	14	3	11.092	0.011	0.204
**177**	TG	CTG	197	12	7	13	2	6	5	6	11	8.957	0.030	0.221
215	TG	CTG	226	19	0	10	5	9	2	16	1	9.404	0.024	0.073
**251**	TG	CTG	248	10	9	13	2	6	5	6	11	8.795	0.032	0.347
**315**	TG	CTG	327	19	0	14	1	11	0	13	4	8.155	0.043	0.193
**440**	TA	CTG	78	13	6	13	2	5	6	6	11	10.269	0.016	0.248
**457**	TA	CTG	91	17	2	11	4	6	5	16	1	8.240	0.041	0.236
**458**	TA	CTG	92	15	4	15	0	9	2	10	7	8.221	0.042	0.227
**473**	TA	CTG	104	14	5	10	5	8	3	3	14	14.530	0.002	**0.025**
**485**	TA	CTG	116	14	5	3	12	7	4	14	3	15.213	0.002	0.053

**490**	TA	CTG	120	17	2	9	6	11	0	15	2	9.114	0.028	0.227
**505**	TA	CTG	133	14	5	8	7	4	7	4	13	9.858	0.020	0.178
**509**	TA	CTG	137	15	4	7	8	6	5	15	2	8.442	0.038	0.203
**852**	TT	CTG	54	16	3	7	8	9	2	15	2	9.325	0.025	0.194
**928**	TT	CTG	129	14	5	14	1	9	2	8	9	9.293	0.026	0.207
930	TT	CTG	133	19	0	15	0	9	2	17	0	9.582	0.022	0.357
**942**	TT	CTG	148	17	2	7	8	6	5	16	1	13.996	0.003	**0.035**
**980**	TT	CTG	199	16	3	9	6	7	4	17	0	9.670	0.022	0.067
**1049**	TG	CTT	60	16	3	7	8	9	2	14	3	7.857	0.049	0.365
**1064**	TG	CTT	75	4	15	9	6	5	6	3	14	8.623	0.035	0.243
**1065**	TG	CTT	76	17	2	7	8	8	3	16	1	12.544	0.006	0.083
1122	TG	CTT	125	19	0	9	6	10	1	17	0	16.979	0.001	**0.020**
**1131**	TG	CTT	132	16	3	15	0	11	0	12	5	8.115	0.044	0.133
1165	TG	CTT	160	16	3	8	7	8	3	16	1	8.329	0.040	0.213
**1171**	TG	CTT	165	14	0	13	2	11	0	7	10	14.087	0.003	**0.024**
**1185**	TG	CTT	176	19	5	13	2	11	0	8	9	21.103	<0.001	**0.002**
**1204**	TG	CTT	189	19	0	12	3	8	3	17	0	9.582	0.022	0.096
**1220**	TG	CTT	201	16	3	7	8	7	4	16	1	11.097	0.011	0.116
**1252**	TG	CTT	229	19	0	12	3	11	0	17	0	9.878	0.020	0.275
**1262**	TG	CTT	238	19	0	10	5	10	1	16	1	10.245	0.017	0.126
**1309**	TG	CTT	281	18	1	9	6	8	3	16	1	9.442	0.024	0.170
**1367**	TG	CTT	357	13	6	13	2	9	2	6	11	11.294	0.010	0.146
1392	TA	CTT	51	8	11	12	3	7	4	14	3	8.212	0.042	0.244
1405	TA	CTT	61	16	3	7	8	10	1	11	6	8.363	0.039	0.232
1441	TA	CTT	92	11	8	4	11	8	3	13	4	9.410	0.024	0.312
**1466**	TA	CTT	114	5	14	5	10	10	1	8	9	12.892	0.005	**0.040**
**1484**	TA	CTT	128	9	10	13	2	6	5	4	13	12.924	0.005	0.076
**1488**	TA	CTT	131	5	14	10	5	2	9	4	13	9.708	0.021	0.250
**1539**	TA	CTT	180	13	6	9	6	9	2	17	0	8.564	0.036	0.192
**1563**	TA	CTT	205	12	7	10	5	11	0	16	1	9.526	0.023	0.182
**1564**	TA	CTT	206	14	5	5	10	9	2	16	1	15.430	0.001	0.063

**1596**	TA	CTT	238	8	11	12	3	6	5	5	12	8.847	0.031	0.133
1618	TA	CTT	263	18	1	14	1	7	4	11	6	8.770	0.033	0.262
**1640**	TA	CTT	293	17	2	13	2	11	0	10	7	9.647	0.022	0.134
1807	TT	CTT	179	19	0	15	0	11	0	14	3	8.345	0.039	0.279
1954	TG	CAA	134	17	2	15	0	11	0	11	6	11.531	0.009	0.104
**2040**	TG	CAA	214	19	0	15	0	9	2	17	0	9.582	0.022	0.357
2133	TA	CAA	82	11	8	9	6	11	0	17	0	14.913	0.002	**0.009**
**2151**	TA	CAA	96	2	17	10	5	3	8	2	15	16.428	0.001	**0.021**
**2168**	TA	CAA	108	19	0	11	4	8	3	17	0	10.928	0.012	**0.026**
2170	TA	CAA	109	19	0	12	3	11	0	17	0	9.878	0.020	0.233
**2212**	TA	CAA	143	19	0	15	0	9	2	17	0	9.582	0.022	0.357
**2213**	TA	CAA	144	7	12	12	3	7	4	5	12	10.416	0.015	0.111
**2215**	TA	CAA	146	11	8	8	7	7	4	17	0	10.667	0.014	**0.040**
**2265**	TA	CAA	190	19	0	10	5	11	0	16	1	13.098	0.004	0.096
**2311**	TA	CAA	229	19	0	15	0	9	2	17	0	9.582	0.022	0.175
2331	TA	CAA	250	19	0	15	0	9	2	17	0	9.582	0.022	0.369
2338	TA	CAA	257	19	0	15	0	9	2	17	0	9.582	0.022	0.357
2469	TT	CAA	57	19	0	12	3	11	0	17	0	9.878	0.020	0.275
2477	TT	CAA	65	18	1	14	1	10	1	11	6	8.231	0.041	0.286
**2493**	TT	CAA	78	13	6	8	7	7	4	2	15	13.354	0.004	**0.031**
2499	TT	CAA	86	13	6	15	0	11	0	14	3	9.007	0.029	0.095
**2619**	TA	CAC	55	16	0	10	2	8	3	16	0	8.433	0.038	0.067
2780	TA	CAC	207	16	0	12	0	9	2	16	0	8.302	0.040	0.186
